# Exploring Agronomic and Physiological Traits Associated With the Differences in Productivity Between Triticale and Bread Wheat in Mediterranean Environments

**DOI:** 10.3389/fpls.2019.00404

**Published:** 2019-04-05

**Authors:** Ana María Méndez-Espinoza, Sebastián Romero-Bravo, Félix Estrada, Miguel Garriga, Gustavo A. Lobos, Dalma Castillo, Iván Matus, Iker Aranjuelo, Alejandro del Pozo

**Affiliations:** ^1^Centro de Mejoramiento Genético y Fenómica Vegetal, Facultad de Ciencias Agrarias, Universidad de Talca, Talca, Chile; ^2^Facultad de Ciencias Agrarias y Forestales, Universidad Católica del Maule, Curicó, Chile; ^3^Núcleo Científico Multidisciplinario-DI, Universidad de Talca, Talca, Chile; ^4^CRI-Quilamapu, Instituto de Investigaciones Agropecuarias, Chillán, Chile; ^5^Instituto de Agrobiotecnología, Consejo Superior de Investigaciones Científicas, Universidad Pública de Navarra, Navarra, Spain

**Keywords:** cereals, grain yield, leaf gas exchange, chlorophyll fluorescence, photosynthesis

## Abstract

In Mediterranean climates soil water deficit occurs mainly during the spring and summer, having a great impact on cereal productivity. While previous studies have indicated that the grain yield (GY) of triticale is usually higher than bread wheat (*Triticum aestivum* L.), comparatively little is known about the performance of these crops under water-limited conditions or the physiological traits involved in the different yields of both crops. For this purpose, two sets of experiments were conducted in order to compare a high yielding triticale (cv. Aguacero) and spring wheat (cvs. Pandora and Domo). The first experiment, aiming to analyze the agronomic performance, was carried out in 10 sites located across a wide range of Mediterranean and temperate environments, distributed between 33°34′ and 38°41′ S. The second experiment, aiming to identify potential physiological traits linked to the different yields of the two crops, was conducted in two Mediterranean sites (Cauquenes and Santa Rosa) in which crops were grown under well-watered (WW) and water-limited (WL) conditions. The relationship between GY and the environmental index revealed that triticale exhibited a higher regression coefficient (Finlay and Wilkinson slope), indicating a more stable response to the environment, accompanied by higher yields than bread wheat. Harvest index was not significantly different between the two cereals, but triticale had higher kernels per spike (35%) and 1000 kernel weight (16%) than wheat, despite a lower number of spikes per square meter. The higher yield of triticale was linked to higher values of chlorophyll content, leaf net photosynthesis (An), the maximum rate of electron transport (ETRmax), the photochemical quantum yield of PSII [Y(II)] and leaf water-use efficiency. GY was positively correlated with Ci at anthesis and Δ^13^C in both species, as well as with gs at anthesis in triticale, but negatively correlated with non-photochemical fluorescence quenching and quantum yield of non-photochemical energy conversion at grain filling in wheat. These results revealed that triticale presented higher photosynthetic rates that contributed to increase plant growth and yield in the different environments, whereas wheat showed higher photoprotection system in detriment of assimilate production.

## Introduction

Triticale ( × *Triticosecale* Wittmack) is a hybrid originating from a cross between wheat (*Triticum* spp.) and rye (*Secale cereale*). The global cropping area for triticale in 2016 was 4,157,018 ha and the average grain yield (GY) is 3.66 t ha^-1^ (FAO, 2018). The largest producers are Poland (1.40 million ha) followed by Belarus and Germany (0.49 and 0.39 million ha, respectively). While triticale is cultivated on only 23,144 ha in Chile (compared to 236,415 ha for wheat), its average grain yield per hectare (GY) is nearly the same as that obtained for wheat (6.1 and 6.0 t ha^-1^ respectively; ODEPA, 2018). Comparative studies among cereals indicate that the GY of triticale is usually higher than bread (*Triticum aestivum* L.) or durum (*Triticum durum*) wheat. Indeed, field trials conducted in Australia, Spain, Sardinia, Lebanon and Tunisia, have shown that triticale yields tend to be greater than bread or durum wheat (Giunta et al., 1993; López-Castañeda and Richards, 1994; Villegas et al., 2010). In the high-yielding environments of the United Kingdom, the average GY of triticale cultivars was also larger than wheat cultivars (Roques et al., 2017).

The higher yield of triticale has been attributed to higher radiation use efficiency (RUE), greater biomass at anthesis and maturity, and a larger number of grains per spike compared to bread wheat (Estrada-Campuzano et al., 2012). Also, triticale exhibited higher biomass, RUE and stomatal conductance (gs) compared to durum wheat under Mediterranean conditions (Motzo et al., 2015). In addition, Planchon (1979) reported higher net photosynthesis (An) and water use efficiency (WUE) in triticale compared to bread and durum wheat plants grown in pots under natural conditions, and Winzeler et al. (1989) reported higher dry matter (DM) accumulation in triticale and lower leaf respiration rates than wheat under controlled environment conditions.

In Mediterranean climates, soil water deficit occurs mainly during the spring and summer, which has a great impact on cereal productivity (Trnka et al., 2014; Hochman et al., 2017). Thus, under rain-fed conditions, the grain filling stage of cereals generally occurs under drought stress (Stanca et al., 2003; Dolferus et al., 2013; Sanchez-Bragado et al., 2014; del Pozo et al., 2016; Hochman et al., 2017), which limits photosynthesis and the production of photosynthetic assimilates that are directly transferred to the grain (Schnyder, 1993). The assimilates necessary for grain filling are provided by photosynthesis in the leaves and spike (Tambussi et al., 2007; Maydup et al., 2010; Sanchez-Bragado et al., 2014), and the redistribution of stored reserves in vegetative tissues during the pre- and/or post-anthesis stages that are translocated to the growing grains (Schnyder, 1993; Yang and Zhang, 2006). Consequently, differences in leaf gas exchange and the performance of the photosynthetic apparatus during anthesis and grain filling may explain the greater productivity of triticale compared to wheat in Mediterranean environments.

At leaf level, leaf water potential (Ψ), net CO_2_ assimilation (An), stomatal conductance (gs), and transpiration (E) decrease under water-limited conditions, leading to changes in instantaneous water-use efficiency (An/E) and intrinsic water-use efficiency (An/gs) in cereals (Tambussi et al., 2007; Sikder et al., 2015; Liu et al., 2017). Also, drought stress can affect the performance of Photosystem II and the electron transport chain, leading to a non-stomatal limitation of photosynthesis (Chaves and Oliveira, 2004). Chlorophyll *a* fluorescence variables and derived parameters obtained from dark-adapted and light-exposed leaves have been used to assess changes in the photosynthetic apparatus as a result of stress (Beer and Björk, 2000; Fracheboud and Leipner, 2003; Harbinson and Rosenqvist, 2003; Estrada et al., 2015). Indeed, differences in the activity of the photosynthetic apparatus among 10 genotypes of triticale have been reported previously, but only for plants grown under well-watered conditions in a glasshouse (Hura et al., 2009). However, to the best of our knowledge, direct comparisons of chlorophyll parameters between wheat and triticale grown in Mediterranean environments under well-watered and water-limited conditions have not been reported.

Carbon isotope discrimination (Δ^13^C) is a valuable trait that integrates the impact of growing conditions on the stomatal opening and carbon assimilation in a large period of time (Monneveux et al., 2006). It also provides indirect information about the efficiency of the water used by the crop (Araus et al., 2008; Blum, 2009). Δ^13^C in kernels can be positive or negatively correlated with GY depending on soil water availability [del Pozo et al., 2016 (for wheat); Munjonji et al., 2017 (for triticale)]. Surprisingly, direct comparison of Δ^13^C in kernels between wheat and triticale, in field grown plants, has not been reported, so far.

This article reports the results of two sets of experiments aiming to compare the performance of a high yielding triticale (cv. Aguacero) and spring wheat (cvs. Pandora and Domo) in a wide range of environments. The objectives of this work were to: (i) evaluate the productivity of triticale and wheat in Mediterranean and temperate environments of central-south of Chile; and (ii) study the performance of the leaf photosynthetic characteristics in the two species, during the grain filling period, and its relationship with agronomic traits, in two contrasting Mediterranean environments, under well-watered and water-limited conditions.

## Materials and Methods

### Plant Material, Growing Conditions, and Experimental Design

In this investigation, high yielding triticale (cv. Aguacero) and spring wheat (cvs. Pandora and Domo) were studied in two sets of field experiments.

#### Experiment 1

Field trials comparing top high yielding cultivars of spring triticale (cv. Aguacero-INIA) and spring bread wheat (Pandora-INIA and Domo-INIA) were conducted at 10 different localities between 33°34′ and 38°41′ S (in 2004 and 2005), representing the wide range of Mediterranean and temperate environments where bread wheat is cultivated in Chile. The date of sowing for each experiment was as recommended for each locality and it ranged from the end of May to the middle of September. The seed rate was 20 g m^-2^ for all cultivars and the plot size was 2 m × 1 m, with the experimental design at each site being randomized block. Crop management included fertilization and weed control in order to provide the optimum growing conditions to all cultivars. Also, irrigation was used in some of the localities. These trials are regularly conducted by INIA for testing cultivars used by farmers and new advanced lines.

#### Experiment 2

Triticale cv. Aguacero and bread wheat cv. Pandora were evaluated at two Mediterranean sites, Cauquenes (35°58′ S, 72°17′ W; 177 m.a.s.l.) and Santa Rosa (36°32′ S, 71°55′ W; 220 m.a.s.l.), under well-watered (WW) and water-limited (WL) conditions, in 2015 and 2016. In 2014 the trial was conducted only at Santa Rosa under WW condition. Cauquenes corresponds to the Mediterranean drought-prone area of Chile and precipitation was 580 and 430 mm in 2015 and 2016, respectively; the soil correspond to a granitic classified as Ultic Palexeralfs. Santa Rosa is an irrigated area and the annual precipitation was 979 and 485 mm in 2015 and 2016, respectively; the soil was a sandy loam, humic haploxerand (Andisol). The WL condition corresponded to rainfed at Cauquenes and Santa Rosa. For the WW condition, sprinkler irrigation was used in Cauquenes and furrow irrigation in Santa Rosa: two to four irrigations after the flag leaf stage (Z37). The sowing rate was 20 g m^-2^ and sowing dates in both years were 18 May at Cauquenes and 29 July at Santa Rosa. Plots consisted of five rows of 2 m in length and 0.2 m distance between rows, in a randomized block design with four replications. Plots received a complete fertilization consisting of: 260 kg ha^-1^ of ammonium phosphate (46% P_2_O_5_ and 18% N), 90 kg ha^-1^ of potassium chloride (60% K_2_O), 200 kg ha^-1^ of sul-po-mag (22% K_2_O, 18% MgO and 22% S), 10 kg ha^-1^ of boronatrocalcite (11% B) and 3 kg ha^-1^ of zinc sulfate (35% Zn). During tillering an extra 153 kg ha^-1^ of N was applied. Weeds were controlled with recommended herbicides and no fungicides were needed.

Leaf sampling was always carried out in leaves in which gas exchange analyses and chlorophyll fluorescence analyses were determined. The leaf samples were plunged immediately into liquid nitrogen and stored at -80°C, for later chlorophyll and proline determinations.

### Agronomic Evaluations

At maturity, the number of spikes per m^2^ (SM2) was determined in a 1 m length section of an inside row of the plot. The aboveground DM and grain biomass was determined in the same 1 m length. Then, harvest index (HI) was calculated and the number of kernels per spike (KS) and 1000 kernel weight (TKW) were determined from the spikes harvested. Plant height (PH) was measured from the base of the plant to the top of the spike (without awns). Finally, GY was estimated from 2 m^2^ in Experiment 1 and from the 1 m length in Experiment 2.

### Leaf Water Potential, Leaf Gas Exchange, and Chlorophyll *a* Fluorescence

Physiological traits were determined at Cauquenes and Santa Rosa during 2014–2016. Leaf water potential, gas exchange, and chlorophyll fluorescence were evaluated on three flag leaves per plot, at anthesis and in grain filling. Leaf water potential (Ψ_L_) was determined in the three flag leaves placed together in a pressure chamber (PMS Instrument, Co., United States).

An, gs, internal CO_2_ concentration (Ci), and transpiration (E) were determined at light saturation, using a portable open system infra-red gas analyzer (CIRAS-2 model, PP Systems, Amesbury, MA, United States) with a 0.250 L min^-1^ flow rate, 380 ppm CO_2_ and leaf temperature at 25°C. Measurements were performed between 12:00 and 16:00 on sunny days at a photon flux density of 1,500 mmol m^-2^ s^-1^, using a broad leaf cuvette (1.7 cm^2^ of leaf area). The instantaneous WUE was calculated as An/E and the intrinsic WUE as An/gs.

Chlorophyll fluorescence of dark and light-acclimated leaves (same leaf samples as used for the leaf gas exchange) was measured with a portable PAM-2500 fluorometer (Walz, Germany) with Leaf-Clip Holder 2030-B (measurement angle 60°). First, each leaf was dark-acclimated for 20 min using a dark leaf clip (DLC-8) in order to determine the minimum (Fo) and maximum (Fm) fluorescence in the dark-adapted state, and the maximum photochemical quantum yield of PSII [Fv/Fm = (Fm – Fo)/Fm]. With the equipment programmed to measure in amplitude modulated pulse mode, the clip was opened and after 30 min of light acclimation, fast light curves (FLCs) were developed considering 10 pulses every 6 s, which increased from 0 to 2000 μmol m^-2^ s^-1^. After each light level, the equipment delivered a light pulse in far-red for 3 s and then a saturation pulse of actinic light (16,500 μmol m^-2^ s^-1^) in order to determine the minimum (Fo′) and maximum (Fm′) chlorophyll fluorescence yield when PSII reaction centers were in the open state. Using an FLC light intensity of 1,500 μmol m^-2^ s^-1^, the following parameters were calculated: Y(II): effective photochemical quantum yield of PSII [(Fm′-F)/Fm′], where F is the fluorescence shortly before a saturating pulse; Y(NPQ): quantum yield of non-photochemical energy conversion in PSII due to down-regulation of the light-harvesting function; Y(NO): quantum yield of non-photochemical energy conversion in PSII other than that caused by down-regulation of the light-harvesting function; and NPQ: non-photochemical fluorescence quenching [(Fm/Fm′)-1]. In addition, the fitting of the FLC according to Eilers and Peeters (1988) allowed determination of the following parameters: Alpha: initial slope of the light curve, related to the maximum photosynthetic yield; ETRmax: maximum rate of electron transport; and IK: the PAR value at the intersection of alpha and ETRmax.

### Chlorophyll and Proline Determination

The same flag leaves used for the non-destructive physiological evaluations were collected and stored at -80°C until use. The leaf tissue was ground with liquid nitrogen and 100 mg of each sample was taken for chlorophyll and proline determination. The pigments were extracted in 1.5 ml *N,N*-dimethylformamide and stored in the dark at 4°C for 48 h (Moran and Porath, 1980). Absorbance of the extracts was measured at 664.5 and 647.0 nm using the simultaneous equations described by Inskeep and Bloom (1985). Concentrations of chlorophyll *a* (Chla) and *b* (Chlb) were expressed on a leaf weight basis (mg g FW^-1^). In addition, chlorophyll content was measured using a DUALEX sensor (Dualex Scientific, Force A, France) as a non-destructive measurement.

Proline concentration was determined according to the method of Bates et al. (1973) with minor modifications. The extraction was carried out with 2 ml of 3% 5-sulfosalicylic acid. This mixture was centrifuged at 7000 rpm for 20 min and the supernatant was obtained. Acetic acid (1 ml) and 1 ml of ninhydrin reagent [ninhydrin 2.5% (w/v), acetic acid 60% (v/v), orthophosphoric acid 23.5% (v/v)] were added to 1 ml of supernatant. The mixture was kept in a hot water bath at 100°C for 45 min and placed on ice for 30 min. After that, 1 ml of toluene was added with the mixture being shaken for 1 min and centrifuged at 7000 rpm for 10 min. The organic phase was collected, and the proline concentration was quantified at 520 nm from a standard curve using L-proline. For the determinations, a UV/VIS T80+ (PG Instruments, United Kingdom) spectrophotometer and Eppendorf 5810R (Eppendorf, Germany) and Heraeus Fresco 17 (Thermo Scientific, United States) centrifuges were used.

### Stomatal Size and Density

For both wheat and triticale, stomatal characterization was performed on the abaxial surface of mature flag leaves (sections from the middle to avoid differential thickness along the leaf), during the 2014 (WW) and 2015 (WW and WL) seasons in Santa Rosa. The characterization process consisted of removal of leaf hairs with transparent adhesive tape and coating the leaf with clear varnish (Bacelar et al., 2006; Guerfel et al., 2009). The dried stomatal impressions were carefully detached with adhesive tape attached to a slide (Ennajeh et al., 2010) and then three photographs were taken with the microscope (Motic BA-310, China). The photographs were analyzed with the Matlab program, while the Motic Images Plus 2.0ML program was used to evaluate stomatal size. The number of stomata was counted in 1.27 mm^2^, equivalent to the diameter of the observed field of the image. For size, five stomata were selected in each image and length and width determination were performed.

### Carbon Isotope Discrimination

The carbon (^13^C/^12^C) isotope ratio in kernels was determined using an elemental analyzer (ANCA-SL, PDZ Europa, United Kingdom) coupled with an isotope ratio mass spectrometer, at the Laboratory of Applied Physical Chemistry at Ghent University (Belgium). The ^13^C/^12^C ratios were expressed as carbon isotope composition: δ^13^C = (((^13^C/^12^C)_sample_/(^13^C/^12^C)_standard_) - 1), where sample refers to plant material and standard to the laboratory standards that have been calibrated against international standards from Iso-Analytical (Crewe, Cheshire, United Kingdom). The precision of δ^13^C analyses was 0.3‰. Further, the carbon isotope discrimination (Δ^13^C) in kernels was calculated as: Δ^13^C (‰) = (δ^13^C_a_ - δ^13^C_p_)/[1+ (δ^13^C_p_)/1000], where a and p refer to air and the plant, respectively (Farquhar et al., 1989). δ^13^C_a_ from the air was taken as -8.0‰.

### Statistical Analysis and Calculations

For physiological and yield-related traits, differences among genotypes (G) and environments (E) were determined through analysis of variance (ANOVA) using Statgraphics Centurion XVII. In the ANOVA, site and water treatments were considered as environments. Regression analyses were performed between the GY of each cultivar and the mean GY of cultivars in each environment (environmental index; Finlay and Wilkinson, 1963). The regression coefficient (Finlay and Wilkinson slope) is a measure of yield adaptability (Calderini and Dreccer, 2002). For the comparison of regression lines, An, gs, and E were linearized with a logarithmic transformation.

For each trait (T), the relative differences (%) were calculated considering water regimes [(T_WW_ –T_WL_/T_WW_)] and species [(T_trit_ – T_wh_)/T_trit_]. Also, correlation and regression analysis were performed between physiological and agronomic traits. Furthermore, principal component analysis (PCA) was carried out using the physiological and agronomic traits of both water regimes and growing seasons (2015 and 2016).

## Results

### Grain Yield and Its Components

**FIGURE 1:**
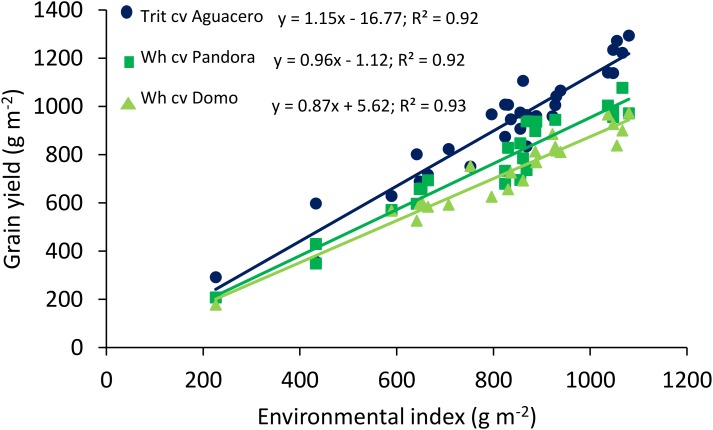
Relationships between the environmental index and grain yield of spring wheat (cv. Pandora-INIA) and triticale (cv. Aguacero-INIA). Data are from 10 localities in 2004 and 2005 (eight Mediterranean and two temperate areas), and two localities (Cauquenes and Santa Rosa), under well-watered and water-deficit conditions, in 2014, 2015, and 2016. Also, data from Mellado et al. (2005) comparing triticale cv. Aguacero-INIA and the spring wheat cv. Domo at nine localities in 2001 and 2002 were included in the analysis. The comparison of the regression lines indicated significant difference among the slopes (*P* < 0.01) and intercepts (*P* < 0.0001).

**FIGURE 2:**
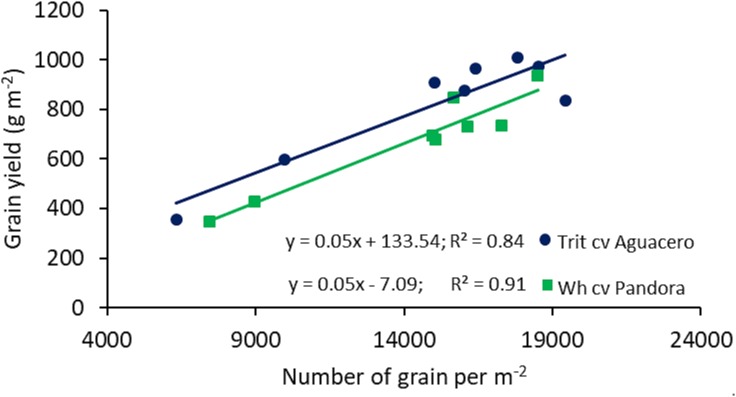
Relationships between the number of grains per m^-2^ and grain yield of spring wheat (cv. Pandora-INIA) and triticale (cv. Aguacero-INIA). Data are from two localities (Cauquenes and Santa Rosa), under well-watered and water-deficit conditions, in 2014, 2015, and 2016. The comparison of the regression lines indicated not significant difference between the slopes (*P* = 0.7359), but significant difference between intercepts (*P* < 0.001).

The relationship between GY and the environmental index, determined in different locations in central Chile, revealed significant differences among the slopes (*P* < 0.01) and intercept (*P* < 0.001); triticale exhibited a higher regression coefficient (Finlay and Wilkinson slope), indicating a more stable response to the environment, accompanied by higher yields than bread wheat, even in extreme environments where the average yield was about 200 g m^-2^ (Figure 1). The linear relationship between GY and the number of grains per m^2^ showed a higher GY in triticale than wheat at any level of grain number (*P* < 0.001 for both cereals) (Figure 2), as a consequence of the larger grain size in triticale. The comparison of the regression lines do not shows differences in the slopes, but it was significant between the intercepts.

The evaluations conducted at Cauquenes and Santa Rosa under WW and WL conditions in 2014, 2015 and 2016, revealed that triticale produced on average 21.6% more aboveground biomass and had a 20.2% larger GY than bread wheat (Table 1). The HI was not significantly different (*P* > 0.05) between the two cereals, but triticale had significantly higher (*P* < 0.001) KS (35%) and TKW (16%) than wheat, even though lower SM2 number (Table 1). The environmental effect was highly significant (*P* < 0.001) for all agronomic traits and the G × E interaction was significant for TKW (Table 1). Under rainfed conditions in Cauquenes, the biomass production and GY were exceptionally high in 2015 (biomass: 1872 and 1745 g m^-2^; GY: 835 and 736 g m^-2^ in triticale and wheat, respectively), due to the large amount of precipitation during October and November, and therefore the differences between WW and WL conditions were minor. PH was significantly different between the two crops (*P* > 0.001) and environments (*P* > 0.000); triticale was on average 21.1% higher than wheat, and this pattern was observed in all the environments (Table 1).

**Table 1:** Above-ground biomass (AB; g m^-2^), grain yield (GY; g m^-2^), harvest index (HI), number of spikes per m2 (SM2), 1000 kernel weight (TKW; g), kernels per spike (KS), plant high (PH), and carbon isotope discrimination (Δ^13^C) for triticale (Trit) cv. Aguacero and wheat (Wh) cv. Pandora, under well-watered (WW) and water-limited (WL) conditions at Santa Rosa (SR) (2014, 2015, and 2016) and Cauquenes (Cau) (2015 and 2016).

Environment	Water regime	Specie	AB	GY	HI	SM2	TKW	KS	PH	Δ^13^C
SR 2014	WW	Trit	-	1138	-	408	-	-	116	-
		Wh	-	956	-	497	-	-	89	-
SR 2015	WW	Trit	2159	1008	0.46	312	56.66	57	118	19.10
		Wh	1493	733	0.49	345	45.43	47	89	19.04
	WL	Trit	2084	874	0.46	306	55.00	52	115	18.96
		Wh	1449	680	0.47	386	45.24	39	88	18.26
CAU 2015	WW	Trit	2033	964	0.48	252	58.98	66	116	16.58
		Wh	2010	938	0.47	336	50.90	55	101	17.45
	WL	Trit	1872	835	0.45	271	43.62	72	124	16.61
		Wh	1745	736	0.42	370	44.10	45	104	17.28
SR 2016	WW	Trit	2089	907	0.44	263	60.35	57	115	16.84
		Wh	1971	847	0.43	411	54.75	38	91	16.43
	WL	Trit	2364	974	0.41	332	52.77	56	116	16.24
		Wh	1671	694	0.42	377	46.49	40	90	16.55
CAU 2016	WW	Trit	1678	598	0.36	240	60.19	41	109	15.69
		Wh	1293	430	0.33	286	47.94	31	90	15.50
	WL	Trit	1176	356	0.31	183	56.79	35	100	13.16
		Wh	1079	349	0.33	267	47.15	28	81	13.67
Mean		Trit	1932	850	0.42	286	55.54	54	114	16.65
		Wh	1589	707	0.42	364	47.75	40	91	16.77
ANOVA	G		^∗∗∗^	^∗∗∗^	n.s.	^∗∗∗^	^∗∗∗^	^∗∗∗^	^∗∗∗^	n.s.
	E		^∗∗∗^	^∗∗∗^	^∗∗∗^	^∗∗∗^	^∗∗∗^	^∗∗∗^	^∗∗∗^	^∗∗∗^
	G × E		n.s.	n.s.	n.s.	n.s.	^∗^	n.s.	n.s.	^∗∗^


*G is genotype, E is environment (year, site, and water regime) and G × E is the genotype × environment interaction. ^∗^*P* < 0.05; ^∗∗^*P* < 0.01; ^∗∗∗^*P* < 0.001; n.s., no significant difference, *P* > 0.05*.

**FIGURE 3:**
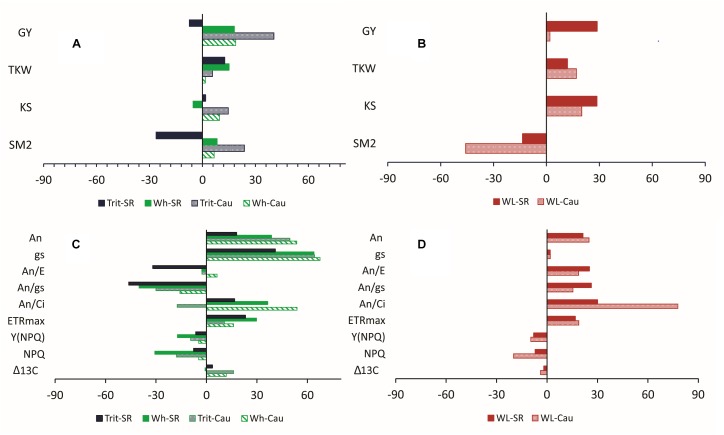
Relative differences (RDs) of agronomic **(A,B)** and physiological **(C,D)** traits (T) considering water regimes (WW and WL; RD = [(T_WW_ – T_WL_)/T_WW_) × 100] **(A,C)** and species (triticale and wheat; RD = [(T_trit_ – T_wh_)/T_trit_) × 100] **(B,D)** at Cauquenes (Cau) and Santa Rosa (SR) in 2016. For the physiological traits, excepting Δ^13^C, the values are mean of measurements conducted at anthesis and grain filling stages. An: leaf net photosynthesis (μmol CO_2_ m^-2^ s^-1^); gs: stomatal conductance (mmol m^-2^ s^-1^); E: transpiration rate (mmol m^-2^ s^-1^); An/E: instantaneous water use efficiency; An/gs: intrinsic water use efficiency; An/Ci: apparent carboxylation efficiency; ETR_max_: maximum rate of electron transport; Y(NPQ): quantum yield of non-photochemical energy conversion in PS II due to down-regulation of the light-harvesting function; NPQ: non-photochemical fluorescence quenching; GY: grain yield; TKW: 1000 kernel weight; KS: kernels per spike; SM2: number of spikes per m2; Δ^13^C: carbon isotope discrimination.

In 2016, the year where the water deficit was more pronounced, the relative differences between water regimes (WW and WL) for agronomic traits (GY, SM2, KS, and TKW) were larger in triticale, particularly at Cauquenes (Figure 3A), indicating that water deficit had a greater impact in triticale compared to wheat. However, the difference in agronomic traits between the two species under WL conditions of Santa Rosa and Cauquenes indicated that triticale had higher GY, TKW, and KS than wheat, but lower SM2 (Figure 3B).

### Physiological Traits

**FIGURE 4:**
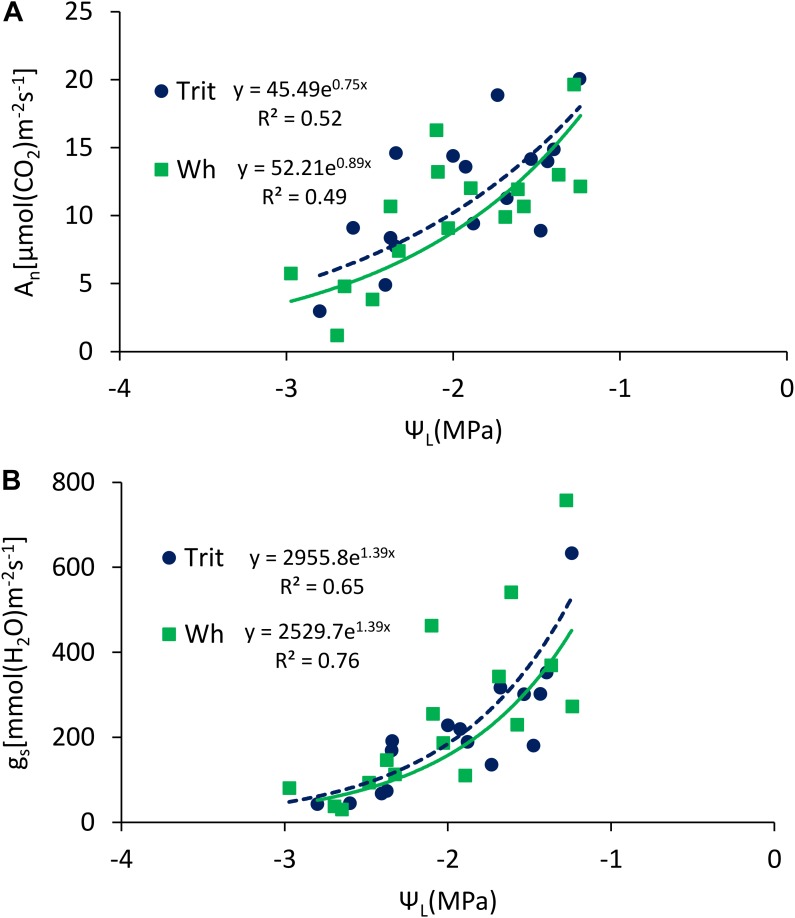
Relationships between leaf water potential (Ψ_L_) and **(A)** maximum net photosynthesis (A_n_), and **(B)** stomatal conductance (g_s_), determined at anthesis and grain filling of triticale and wheat grown under well-watered (WW) and water-limited (WL) conditions, at Cauquenes and Santa Rosa in 2014, 2015, and 2016. The comparison of the regression lines indicated not significant difference between the slopes (*P* = 0.34; 0.36 for **A,B**), or the intercepts (*P* = 0.57; 0.85 for **A,B**).

**FIGURE 5:**
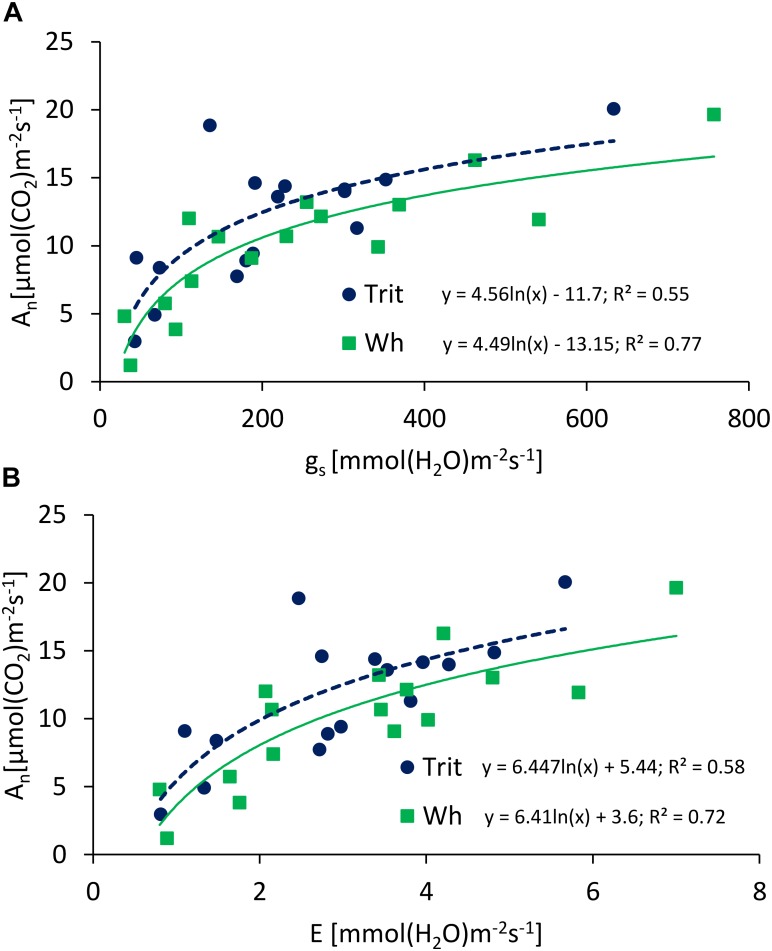
Relationships between maximum net photosynthesis (A_n_) and **(A)** stomatal conductance to CO_2_ (g_s_) and **(B)** transpiration (E), determined at anthesis and grain filling of triticale and wheat grown under well-watered (WW) and water-limited (WL) conditions, at Cauquenes and Santa Rosa in 2014, 2015, and 2016. The comparison of the regression lines indicated significant difference between the slopes (*P* = 0.01; 0.002 for **A,B**), but not significant difference between intercepts (*P* = 0.47; 0.26 for **A,B**).

The Ψ_L_ and E at anthesis and grain filling were not significantly (*P* > 0.05) different between the two cereals, but triticale had on average a 23% higher An at anthesis and 24.7% lower gs than bread wheat (Table 2). The G × E interaction was only significant for gs and Ci at grain filling. The ratios of An/E, An/gs at grain filling, and An/Ci at anthesis and grain filling were superior in triticale (Table 2), particularly under WL conditions (Figure 3D). The environmental effect was highly significant (*P* < 0.001) for all traits and developing stages (Table 2); in 2016, An, gs, and E were severely reduced under WL conditions in both species (Figure 3C), but triticale had higher An and gs, and lower Ci at anthesis than wheat (Table 2). The exponential responses of An and gs to Ψ_L_ were similar in the two cereals (Figure 4), with no statistical differences between the slopes and the intercepts. As expected, positive relationships were found between An vs. gs, and An vs. E, indicating significant higher An and E values per unit of gs in triticale compared to wheat (Figure 5).

**Table 2:** Mean values of leaf water potential and leaf gas exchange determined at anthesis (A) and grain filling (GF; soft dough grain), in triticale (Trit) cv. Aguacero and wheat (Wh) cv. Pandora under well-watered (WW) and water-limited (WL) conditions at Santa Rosa (SR) (2014, 2015, and 2016) and Cauquenes (Cau) (2015 and 2016).

Environment	Water regime	Species	Ψ_L_		An		gs		E		Ci		An/E		An/gs		An/Ci	
			
			A	GF	A	GF	A	GF	A	GF	A	GF	A	GF	A	GF	A	GF
SR 2014	WW	Trit	-1.24	-1.53	20.1	14.2	633	302	5.7	4.0	287	270	3.75	3.85	0.03	0.05	0.07	0.05
		Wh	-1.28	-1.61	19.7	11.9	757	541	7.0	5.8	297	316	2.86	2.12	0.03	0.02	0.07	0.04
SR 2015	WW	Trit	-	-1.68	–	11.3	–	317	–	3.8	–	294	–	3.06	–	0.04	–	0.04
		Wh	–	-1.69	–	9.9	–	343	–	4.0	–	307	–	2.55	–	0.03	–	0.03
	WL	Trit	-1.4	-2.35	14.9	7.7	352	169	4.8	2.7	286	283	3.14	2.83	0.04	0.05	0.05	0.03
		Wh	-1.24	-2.48	12.2	3.8	273	93	3.8	1.8	283	334	3.31	2.32	0.05	0.04	0.04	0.01
Cau 2015	WW	Trit	–	-1.88	–	9.4	–	189	–	3.0	–	268	–	3.18	–	0.06	–	0.04
		Wh	–	-2.03	–	9.1	–	187	–	3.6	–	286	–	2.62	–	0.05	–	0.03
	WL	Trit	-1.43	-2.8	14.0	3.0	302	43	4.3	0.8	275	262	3.71	3.71	0.05	0.07	0.05	0.01
		Wh	-1.37	-2.7	13.0	1.2	369	38	4.8	0.9	286	364	2.86	0.47	0.04	0.01	0.05	0.004
SR 2016	WW	Trit	-1.93	-2	13.6	14.4	219	228	3.5	3.4	251	253	3.91	4.64	0.06	0.07	0.06	0.06
		Wh	-2.09	-2.1	13.2	16.3	255	462	3.4	4.2	259	281	3.89	4.33	0.06	0.04	0.05	0.06
	WL	Trit	-2.34	-2.38	14.6	8.4	191	73	2.8	1.5	236	195	5.7	5.58	0.08	0.11	0.06	0.04
		Wh	-2.38	-2.33	10.7	7.4	146	113	2.2	2.2	247	273	5.02	3.42	0.07	0.07	0.04	0.03
Cau 2016	WW	Trit	-1.73	-1.48	18.9	8.9	135	180	2.5	2.8	101	282	7.79	3.04	0.15	0.05	0.20	0.03
		Wh	-1.89	-1.58	12.0	10.7	110	230	2.1	3.5	123	278	6.42	3.22	0.14	0.05	0.09	0.04
	WL	Trit	-2.6	-2.41	9.1	4.9	45.5	67	1.1	1.3	40	294	8.2	2.92	0.2	0.06	0.25	0.02
		Wh	-2.65	-2.97	4.8	5.7	30.3	80	0.8	1.6	127	294	6.0	3.03	0.16	0.06	0.04	0.02
Mean		Trit	-1.81	-2.05	15.0	9.13	268	174	3.52	2.59	210	267	5.2	3.64	0.09	0.06	0.10	0.04
		Wh	-1.84	-2.17	12.2	8.45	277	231	3.43	3.07	232	304	4.3	2.68	0.08	0.04	0.05	0.03
ANOVA	G		n.s.	n.s.	^∗^	n.s.	n.s.	^∗^	n.s.	n.s.	^∗∗^	^∗∗∗^	^∗^	^∗∗∗^	n.s.	^∗∗∗^	^∗∗∗^	^∗^
	E		^∗∗∗^	^∗∗∗^	^∗∗^	^∗∗∗^	^∗∗∗^	^∗∗∗^	^∗∗∗^	^∗∗∗^	^∗∗∗^	^∗∗∗^	^∗∗^	^∗∗^	^∗∗∗^	^∗∗∗^	^∗∗∗^	^∗∗∗^
	G × E		n.s.	n.s.	n.s.	n.s.	n.s.	^∗^	n.s.	n.s.	n.s.	^∗^	n.s.	n.s.	n.s.	n.s.	^∗∗^	n.s.


*G is genotype, E is environment (year, site, and water regime) and G × E is the genotype × environment interaction. Ψ_*L*_: leaf water potential (MPa); An: leaf net photosynthesis (μmol CO_*2*_ m^-*2*^ s^-*1*^); gs: stomatal conductance (mmol m^-*2*^ s^-*1*^); E: transpiration rate (mmol m^-*2*^ s^-*1*^); Ci: internal CO_*2*_ concentration (mmol mol^-*1*^); An/E: instantaneous water use efficiency; An/gs: intrinsic water use efficiency; An/Ci: apparent carboxylation efficiency. ^∗^*P* < 0.05; ^∗∗^*P* < 0.01; ^∗∗∗^*P* < 0.001; n.s., no significant difference, *P* > 0.05.*

Δ^13^C in kernels was not significantly different between the two species but the G × E interaction was significant (*P* < 0.01) different (Table 1). The WL condition in Cauquenes, 2016 reduced Δ^13^C by 16 and 12% in triticale and wheat, respectively (Figure 3C).

Among the chlorophyll fluorescence parameters, triticale had significantly lower values of Fo at grain filling (*P* < 0.001) and Fm at anthesis (*P* > 0.05), but higher values of ∼Fo′ and Fm′ at anthesis (*P* < 0.05) (Table 3). The parameters for light-acclimated leaves, IK, ETRmax, and Y(II) were significant higher (*P* < 0.001) and Y(NPQ) was significantly lower (*P* < 0.05) in triticale at anthesis and grain filling (Figure 3D and Table 4). Under WL conditions in 2016, ETRmax was less effected in triticale, and also Y(NPQ) and NPQ increase more than in wheat, indicating better photochemical efficiency in triticale (Figure 3C).

**Table 3:** Mean values of chlorophyll index and chlorophyll fluorescence parameters determined at anthesis (A) and grain filling (GF; soft dough grain) in triticale (Trit) cv. Aguacero and wheat (Wh) cv. Pandora under well-watered (WW) and water-limited (WL) conditions at Santa Rosa (SR) (2014, 2015, and 2016) and Cauquenes (Cau) (2015 and 2016).

Environment	Water regime	Species	Chl index		Fo		Fm		Fv/Fm		∼Fo′		Fm′	
								
			A	GF	A	GF	A	GF	A	GF	A	GF	A	GF
SR 2014	WW	Trit	47.76	44.62	1.64	1.68	6.47	6.56	0.75	0.74	1.3	1.43	2.63	2.87
		Wh	43.54	32.34	1.61	1.79	6.46	6.32	0.75	0.71	1.22	1.49	2.66	3.03
SR 2015	WW	Trit	–	33.40	–	1.36	–	5.99	–	0.71	–	1.25	–	2.62
		Wh	–	25.58	–	1.55	–	6.66	–	0.77	–	1.32	–	2.72
	WL	Trit	44.14	32.89	1.5	1.34	6.29	6.02	0.7	0.71	1.38	1.18	2.92	2.39
		Wh	40.85	29.85	1.61	1.52	6.82	6.27	0.76	0.75	1.22	1.19	2.61	2.41
Cau 2015	WW	Trit	–	50.32	–	1.49	–	6.19	–	0.7	–	3.01	–	1.37
		Wh	–	38.73	–	1.68	–	6.87	–	0.76	–	3.29	–	1.41
	WL	Trit	52.54	45.91	1.54	1.45	6.82	6.26	0.77	0.7	1.38	1.36	3.14	3
		Wh	42.14	33.67	1.59	1.68	6.85	6.85	0.77	0.75	1.31	1.27	2.95	2.5
SR 2016	WW	Trit	59.06	53.88	1.37	1.33	6.21	6.02	0.78	0.79	1.2	1.1	2.6	2.33
		Wh	52.20	46.41	1.35	1.39	6.67	6.52	0.8	0.79	1.16	1.21	2.69	2.93
	WL	Trit	58.00	46.12	1.38	1.35	6.63	5.88	0.79	0.77	1.2	1.09	2.79	2.09
		Wh	51.19	38.58	1.38	1.45	6.65	6.06	0.79	0.76	1.2	1.13	2.55	2.17
Cau 2016	WW	Trit	49.98	54.34	1.52	1.51	6.85	6.57	0.78	0.77	1.33	1.25	2.78	2.61
		Wh	43.35	42.64	1.5	1.55	6.86	6.67	0.78	0.77	1.12	1.2	2.24	2.3
	WL	Trit	47.66	44.58	1.55	1.45	6.64	6.63	0.77	0.78	1.29	1.14	2.69	2.15
		Wh	42.53	37.18	1.54	1.46	6.86	6.67	0.78	0.78	1.14	1.1	2.35	2.02
Mean		Trit	51.31	45.12	1.5	1.44	6.56	6.24	0.76	0.74	1.3	1.24	2.79	2.56
		Wh	45.12	36.11	1.51	1.56	6.74	6.54	0.78	0.76	1.2	1.26	2.58	2.6
ANOVA	G		^∗∗^	^∗∗∗^	n.s.	^∗∗∗^	n.s.	n.s.	n.s.	n.s.	^∗∗^	n.s.	^∗^	n.s.
	E		^∗∗^	^∗∗∗^	^∗∗∗^	^∗∗∗^	n.s.	n.s.	^∗^	n.s.	^∗^	^∗∗∗^	^∗^	^∗∗∗^
	G × E		n.s.	n.s.	n.s.	n.s.	n.s.	n.s.	n.s.	n.s.	n.s.	n.s.	n.s.	^∗^


*G is genotype, E is environment (year, site, and water regime) and G × E is the genotype × environment interaction. Fo and Fm: minimum and maximum fluorescence in the dark-adapted state, respectively; Fv/Fm: maximum photochemical quantum yield of photosystem II; ∼Fo**′** and Fm**′**: minimum and maximum chlorophyll fluorescence yield, respectively, when the photosystem II reaction centers were in the open state. ^∗^*P* < 0.05; ^∗∗^*P* < 0.01; ^∗∗∗^*P* < 0.001; n.s., no significant difference, *P* > 0.05*.

**Table 4:** Mean values of chlorophyll fluorescence parameters determined at anthesis (A) and grain filling (GF; soft dough grain), of triticale (Trit) cv. Aguacero and wheat (Wh) cv. Pandora under well-watered (WW) and water-limited (WL) conditions at Santa Rosa (SR) (2014, 2015, and 2016) and Cauquenes (Cau) (2015 and 2016).

Environment	Water regime	Species	Alpha		IK		ETRmax		Y(II)		Y(NPQ)		Y(NO)		NPQ	
									
			A	GF	A	GF	A	GF	A	GF	A	GF	A	GF	A	GF
SR 2014	WW	Trit	0.32	0.34	309.7	274.0	98.2	88.1	0.16	0.15	0.50	0.48	0.34	0.37	0.37	1.31
		Wh	0.32	0.34	351.7	189.5	111.2	64.7	0.17	0.11	0.49	0.46	0.34	0.43	0.37	1.12
SR 2015	WW	Trit	–	0.31	–	331.9	–	101.6	–	0.16	–	0.45	–	0.32	–	1.35
		Wh	–	0.33	–	231.9	–	75.1	–	0.12	–	0.52	–	0.36	–	1.51
	WL	Trit	0.30	0.35	333.4	202.0	97.7	68.6	0.16	0.12	0.45	0.52	0.32	0.29	1.29	1.66
		Wh	0.31	0.34	285.7	196.6	86.7	66.7	0.15	0.10	0.53	0.56	0.33	0.34	1.73	1.66
Cau 2015	WW	Trit	–	0.32	–	337.9	–	105.6	–	0.17	–	0.42	–	0.34	–	1.15
		Wh	–	0.33	–	284.0	–	92.3	–	0.14	–	0.45	–	0.41	–	1.12
	WL	Trit	0.30	0.33	453.1	240.6	132.7	78.1	0.20	0.12	0.43	0.45	0.37	0.35	1.23	1.26
		Wh	0.29	0.35	372.0	165.2	105.1	57.6	0.17	0.09	0.48	0.58	0.36	0.33	1.42	1.85
SR 2016	WW	Trit	0.31	0.32	479.8	399.3	147.0	132.9	0.22	0.20	0.46	0.49	0.33	0.31	1.42	1.62
		Wh	0.31	0.32	424.0	372.6	131.0	123.5	0.19	0.18	0.48	0.45	0.33	0.37	1.51	1.17
	WL	Trit	0.33	0.32	401.2	267.2	130.5	84.5	0.19	0.14	0.46	0.55	0.34	0.31	1.42	1.85
		Wh	0.31	0.30	357.3	238.8	109.8	69.1	0.17	0.11	0.52	0.57	0.32	0.32	1.65	1.85
Cau 2016	WW	Trit	0.31	0.31	395.2	468.8	119.7	141.3	0.19	0.22	0.48	0.47	0.33	0.31	1.50	1.53
		Wh	0.29	0.31	395.9	368.1	116.1	109.4	0.19	0.18	0.55	0.54	0.26	0.28	2.16	1.93
	WL	Trit	0.30	0.31	422.2	351.3	125.4	107.8	0.20	0.17	0.48	0.56	0.32	0.27	1.48	2.09
		Wh	0.29	0.33	336.7	274.7	99.4	89.8	0.16	0.15	0.55	0.59	0.29	0.26	1.96	2.32
Mean		Trit	0.31	0.32	399.2	319.2	121.6	100.9	0.19	0.16	0.46	0.49	0.34	0.32	1.24	1.54
		Wh	0.30	0.33	360.5	257.9	108.4	83.1	0.17	0.13	0.51	0.52	0.32	0.35	1.54	1.61
ANOVA	G		n.s.	n.s.	^∗^	^∗∗∗^	^∗∗^	^∗∗∗^	^∗∗^	^∗∗∗^	^∗∗∗^	^∗^	n.s.	^∗^	^∗∗∗^	n.s.
	E		^∗^	^∗^	^∗∗∗^	^∗∗∗^	^∗∗∗^	^∗∗∗^	^∗∗^	^∗∗∗^	n.s.	^∗∗∗^	n.s.	^∗∗∗^	^∗∗∗^	^∗∗∗^
	G × E		n.s.	n.s.	n.s.	n.s.	n.s.	n.s.	n.s.	n.s.	n.s.	n.s.	n.s.	n.s.	n.s.	n.s.


*G is genotype, E is environment (year, site, and water regime) and G × E is genotype × environment interaction. Alpha: initial slope of light curve, related to maximum photosynthetic yield; IK: PAR value of the point of intersection between a horizontal line ERT_*max*_ and the extrapolated initial slope; ETR_*max*_: maximum rate of electron transport; Y(II) = (Fm′-F)/Fm′: effective photochemical quantum yield of photosystem II; Y(NPQ): quantum yield of non-photochemical energy conversion in PS II due to down-regulation of the light-harvesting function; Y(NO): quantum yield of non-photochemical energy conversion in PS II other than that caused by down-regulation of the light-harvesting function; NPQ, non-photochemical fluorescence quenching. ^∗^*P* < 0.05; ^∗∗^*P* < 0.01; ^∗∗∗^*P* < 0.001; n.s., no significant difference, *P* > 0.05.*

Y(NO) and NPQ also differed between the two crops, being lower in triticale at grain filling and anthesis, respectively (Table 4). A significant environmental effect was observed for all chlorophyll fluorescence traits, except for Fm, however the G × E interaction was not significant (Tables 3, 4). The chlorophyll index and concentration were not different between the two crops, but the chlorophyll index and the Chl a:b ratio at grain filling was significantly higher in triticale (*P* < 0.001) (Tables 3, 5). During grain filling, the water-deficit reduced pigments and increased proline content in leaves (Table 5).

**Table 5:** Mean values of chlorophyll index, pigments (mg gFW^-1^) and proline (mg gFW^-1^) determined at anthesis (A) and grain filling (GF; soft dough grain), of triticale (Trit) cv. Aguacero and wheat (Wh) cv. Pandora under well-watered (WW) and water-limited (WL) conditions at Santa Rosa (2014 and 2015) and Cauquenes (2015).

Environment	Water regime	Species	Chl a		Chl b		Chl a:b		Chl T		Proline	
			
			A	GF	A	GF	A	GF	A	GF	A	GF
SR 2014	WW	Trit	1.68	1.94	0.98	1.16	1.71	1.67	2.93	3.42	0.3	0.29
		Wh	1.83	1.67	1.09	1.03	1.72	1.63	3.28	1.09	0.24	0.26
SR 2015	WW	Trit	–	1.17	–	0.91	–	1.29	–	2.1	–	0.46
		Wh	–	0.95	–	0.76	–	1.23	–	2.97	–	0.33
	WL	Trit	1.68	0.86	1.3	0.68	1.29	1.28	2.99	1.54	0.19	0.65
		Wh	1.88	0.60	1.41	0.50	1.29	1.19	3.26	1.71	0.63	0.81
CAU 2015	WW	Trit	–	1.69	–	1.28	–	1.33	–	2.99	–	0.98
		Wh	–	1.91	–	1.46	–	1.31	–	3.38	–	0.59
	WL	Trit	1.90	1.30	1.46	1.00	1.31	1.30	3.38	2.31	0.29	2.67
		Wh	1.76	1.02	1.37	0.83	1.29	1.22	3.15	1.86	0.20	2.51
Mean		Trit	1.75	1.39	1.25	1.01	1.44	1.37	3.10	2.47	0.26	1.01
		Wh	1.83	1.23	1.29	0.91	1.43	1.31	3.23	2.20	0.36	0.9
ANOVA		G	n.s.	n.s.	n.s.	n.s.	n.s.	^∗∗∗^	n.s.	n.s.	n.s.	n.s.
		E	n.s.	^∗∗∗^	^∗∗^	^∗∗∗^	^∗∗∗^	^∗∗∗^	n.s.	^∗∗∗^	n.s.	^∗∗^
		G × E	n.s.	n.s.	n.s.	n.s.	n.s.	n.s.	n.s.	n.s.	n.s.	n.s.


*G is genotype, E is environment (year, site, and water regime) and G × E is genotype × environment interaction. Chlorophyll a (Chl a), chlorophyll b (Chl b), relationship between chlorophyll a and b (Chl a:b), total chlorophyll (Chl T) and proline. ^∗^*P* < 0.05; ^∗∗^*P* < 0.01; ^∗∗∗^*P* < 0.001; n.s., no significant difference, *P* > 0.05*.

The evaluation of stomata morphology on the abaxial surface indicated that triticale had wider stomata, but lower stomatal density (25.3% less) than wheat (Table 6). The length of the stomata showed no differences between cereals. Water-deficit in 2015 reduced the stomatal size in both species.

**Table 6:** Stomatal width (S_W_), stomatal length (S_L_), and stomatal density (ED, number of stomata per square millimeter) at grain filling in triticale cv. Aguacero and bread wheat cv. Pandora, at Santa Rosa under well-watered (WW) conditions in 2014 and under WW and water-limited (WL) conditions in 2015.

Year	Water regime	Species	S_W_ (mm)	S_L_ (mm)	ED (mm^2^)
2014	WW	Tri	26.08	50.36	49.78
		Wh	24.80	50.50	62.95
2015	WW	Tri	33.86	54.81	47.64
		Wh	29.22	52.04	66.32
	WL	Tri	26.14	48.39	46.63
		Wh	25.33	48.00	63.65
Mean		Tri	28.69	51.19	48.02
		Wh	26.45	50.18	64.31
ANOVA		G	^∗∗∗^	n.s.	^∗∗∗^
		E	^∗∗∗^	^∗∗∗^	n.s.
		G × E	^∗∗∗^	n.s.	n.s


*G is genotype, E is environment (year, site, and water regime) and G × E is genotype × environment interaction. ^∗^*P* < 0.05; ^∗∗^*P* < 0.01; ^∗∗∗^*P* < 0.001; n.s., no significant difference, *P* > 0.05.*

### Relationships Between Physiological and Agronomic Traits

Correlation analysis between physiological traits (determined at anthesis and grain filling) and agronomic traits, showed a number of significant correlations, in both triticale and wheat (Table 7). GY was positively (and significant) correlated with Ci at anthesis and Δ^13^C in both species, as well as with gs at anthesis in triticale and fluorescence parameters [∼Fo′, Fm′, Y(NO)] at grain filling in wheat. In addition, GY was negatively correlated with An/gs at anthesis and NPQ at grain filling in both species, An/Ci at anthesis in triticale, and Y(NPQ) at grain filling in wheat. The TKW was positively correlated with An, gs, An/Ci, Ψ_L_, ETRmax, and Y(II) at grain filling in triticale, and with Chl and ETRmax in wheat. KS had a significant and positive correlation with Ci, and a negative correlation with An/gs at anthesis, in both species; also significant correlations with some of the fluorescence parameters and Δ^13^C in wheat. In the case of HI the correlations were in general similar to those observed for GY.

**Table 7:** Correlation matrix between agronomic and physiological traits determined at anthesis (A) and grain filling (GF), in triticale and wheat.

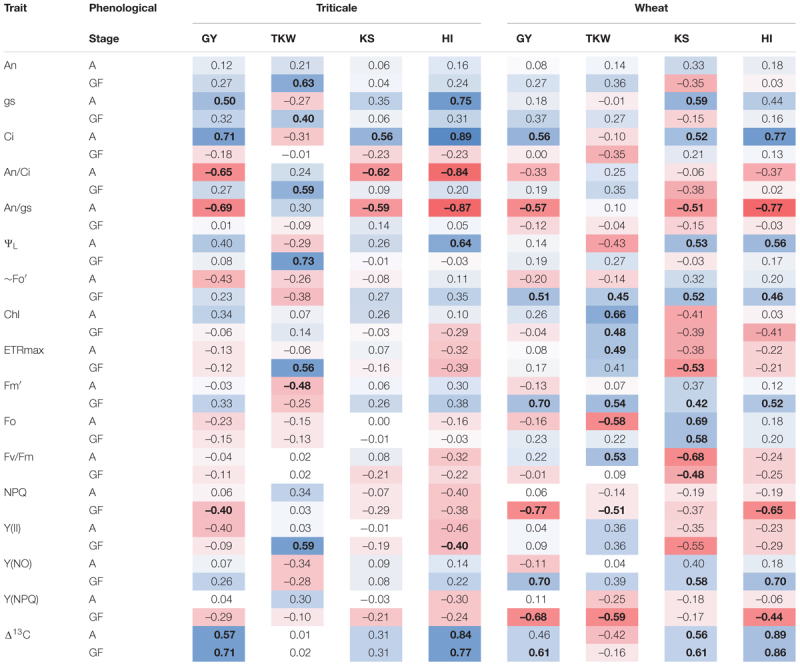


*Data are from two localities (Cauquenes and Santa Rosa), under well-watered and water-limited conditions, during 2015 and 2016. Coefficients of significant correlations (at *P* < 0.05) are in bold characters. Abbreviation as in previous tables*.

The PCA revealed that the first two principal components (PC) explained 57.2% of the observed variability (Supplementary Figure S1). There was a clear separation between years and species; the separation between both species was irrespective of the water regime. The clustering of the year 2016 was mainly based on physiological variables linked with plant water status (Ψ_L_), the net assimilation (An), efficiency in the use of water (An/gs, An/E), photochemical efficiency Y(II), IK, ETRmax, thermal dissipation of the photosynthetic apparatus [Y(NPQ) and NPQ] and chlorophyll content (Chl). In the other hand, the clustering of 2015 was mainly associated by productive variables such as GY, KPS, SM2, HI, AB, and PH (Supplementary Figure S1B).

## Discussion

### Yield and Its Agronomic Components

The comparison between cereals in terms of traits associated with drought tolerance (Sinha et al., 1986; Lonbani and Arzani, 2011; Roohi et al., 2013; Kumar et al., 2014), source limitations (Calderini et al., 2006; Motzo et al., 2013), yield potential (Ugarte et al., 2007; Villegas et al., 2010; Bassu et al., 2011; Estrada-Campuzano et al., 2012; Motzo et al., 2015), molecular markers and gene expression (Kavanagh et al., 2013; Latini et al., 2013), among others, contribute to understand the physiological basis of crop production. In our study, the comparison between the best spring cultivars of triticale and bread wheat indicated that triticale outyielded bread wheat in a wide range of environments (Figure 1). The regression coefficient (Finlay and Wilkinson slope) was significant (*P* < 0.01) larger in triticale (1.15 vs. 0.96 and 0.87 in wheat), indicating greater adaptability. The environmental index is a valuable tool for discriminating cultivars with augmented tolerance to drought stress (Sio-Se Mardeh et al., 2006), but also for genotypes of high yield potential in more favorable environments, like triticale in our study. The higher yield potential of triticale in high yielding environments has also been recorded in 16 sites in the United Kingdom, where the average GY of two triticale cultivars (8.27 and 9.01 t ha^-1^) was higher compared to two wheat cultivars (7.26 and 7.94 t ha^-1^) (Roques et al., 2017). Other studies comparing triticale and durum wheat in drought-prone Mediterranean environments (Australia, Spain, Sardinia, Lebanon, and Tunisia), also revealed the higher GY of triticale (Giunta et al., 1993; López-Castañeda and Richards, 1994; Villegas et al., 2010). However, in a subtropical climate like New Dehli, India, triticale did not outyield bread or durum wheat, with or without irrigation (Sinha et al., 1986).

Agronomic components help to understand the variations in GY associated with genetic improvement, crop management or environmental factors in cereals (Giunta et al., 1993; del Pozo et al., 2012, 2014, 2016). In this study, the higher GY of triticale is explained by greater TKW and KS values (Table 1). Also, the higher KS in triticale was able to compensate for the lower number of spikes per m^2^, compared to wheat. The number of grains per unit area is the major component that determines yield in cereals (Peltonen-Sainio et al., 2007; Slafer et al., 2014) and in the case of triticale and wheat, the average number of grains per m^2^ were similar (14,943 and 14,256 grain m^-2^, respectively). However, the relationship between GY and the number of grains per m^2^ (Figure 2) indicates that the greater TKW of triticale explained the differences in GY between the two cereals. Indeed, spike size and fertility have been demonstrated to contribute more than tillering capacity to the number of grains set per m^2^ (Motzo et al., 2015). In other cases, an increase in the proportion of grains in distal positions (Ferrante et al., 2017) increases the final yield. Finally, HI was similar in both species but, under more severe stress (2016) wheat tended to present higher HI than triticale.

In areas with Mediterranean climates, the grain filling stage of cereals generally occurs under conditions of water scarcity (Dolferus et al., 2013; Sanchez-Bragado et al., 2014), and the effect of precipitation or water availability influences GY via fluctuations in the amount and frequency (Sinha et al., 1986), but the relative impact varies with the intensity of stress and the plant species (Pinheiro et al., 2011). In our study, rainfalls occurred during grain filling (in October–November) in 2015 led to exceptionally high GY (>800 g m^-2^) at Cauquenes, and actually no differences were detected between WW and WL (rainfed) conditions in that year (Table 1). However, in 2016 the water deficit was more severe, particularly at Cauquenes, and this lead to a reduction in GY and its components, in both species.

In this study, the PH of triticale was an overage 25% higher than wheat (Table 1), which agree with other studies (Khan et al., 2015; Motzo et al., 2015; Roques et al., 2017). Indeed, the higher PH of triticale can improve crop competitiveness (Beres et al., 2010), which is consistent with its higher GY and biomass. In addition, higher PH and biomass probably imply greater amount of water-soluble carbohydrate that can be mobilized to the grain, especially under water-limited conditions (Xue et al., 2009).

### Physiological Traits

The assimilates necessary for filling the grain are provided by photosynthesis in the leaves (Evans et al., 1975) and spikes (Tambussi et al., 2007; Maydup et al., 2012), and the redistribution of reserves stored in vegetative tissues during the pre- and/or post-anthesis periods, which are translocated to the growing grains (Schnyder, 1993; Zhang et al., 2006). Triticale had higher leaf net photosynthesis than bread wheat, in all the environments and water conditions tested in our study, which could explain the higher biomass of this species. Moreover, the fact that triticale had higher photosynthetic rates during the grain filling period, especially in the initial stages of grain filling, reveals that the contribution of C assimilated during this stage to grain filling was higher than in wheat. Such a trait also contributed to the increased GY of triticale, through the production of reserve carbohydrates, which are stored in the stem and then mobilized to the grain (Méndez et al., 2011; del Pozo et al., 2012; Stella et al., 2016; Yáñez et al., 2017). On the other hand, our study showed that drought stress impaired photosynthesis and the production of photosynthetic assimilates that are directly transferred to the grain (Schnyder, 1993), as observed in Cauquenes in 2016 where An and GY were severely reduced under WL conditions, but triticale maintained higher rates of phosynthesis at anthesis than wheat (Table 2).

Stomatal opening proved to be a target factor involved in the different photosynthetic performance of triticale and bread wheat plants. Stomata control the balance of gases between the internal leaf environment and the external atmosphere (McAusland et al., 2016) regulating CO_2_ uptake for photosynthesis and transpiration and thus determining plant productivity (Lawson and Blatt, 2014). In addition, the stomatal size and density vary greatly between plant species (Giday et al., 2013) and are influenced by the growing environment (Hetherington and Woodward, 2003; Franks and Beerling, 2009). This was evident in the present study, where a mild water stress at Santa Rosa in 2015 generated a significant reduction in stomatal width and length in both cereals (Table 6).

Leaves with smaller stomata and greater stomatal density respond quickly to changes in water availability (Drake et al., 2013; Giday et al., 2013; Raven, 2014; Buckley, 2017). In fact, negative correlations between stomatal density and size were reported for wheat (Shahinnia et al., 2016; Hughes et al., 2017). In our study, triticale had lower stomatal density (25.3%) and higher response of gs to water availability compared to bread wheat, especially during grain filling, thus increasing the efficiency of water use in leaves (Franks et al., 2015). In fact, An/E and An/gs were higher in triticale compared to wheat (35.8 and 50% respectively, in grain filling) (Table 2), particularly under WL conditions in 2016 (Figure 3C), indicating that for a similar loss of water the amount of photosynthesis was higher in triticale (Figure 5). Also, the apparent carboxylation efficiency (An/Ci) was less affected by WL conditions in triticale (Figure 3C), suggesting greater intrinsic photosynthetic capacity than wheat (Monneveux et al., 2006).

Abiotic stress has adverse effects on the whole plant and on its metabolites, but chloroplasts and proteins are substantially affected by stress factors (Huseynova et al., 2016). Under water-limited conditions the leaf water potential decreased and proline content increased, without significant (*P* < 0.05) differences between the cereals. The larger availability of free proline in drought-affected plants was associated with its role as an osmoregulant that contributes to overcoming leaf dehydration by maintaining negative water potential to avoid water loss (Ramanjulu and Bartels, 2002). In this study, proline content increased during grain filling in both species, associated to the decline in Ψ_L_ (Tables 2, 5).

Also, at the grain filling stage triticale presented a higher Chl index and chl a:b (29.4 and 4.6%, respectively) than wheat. It has been reported that the slow senescence of the leaf canopy (chlorophyll retention) after flowering helps to maintain the process of grain filling (Gous et al., 2013; Kholová et al., 2014). Therefore, triticale seems to maintain higher chlorophyll content and rates of photosynthesis during grain filling than wheat, indicating that triticale has a functional stay-green mechanism (Borrell et al., 2000), which could be related to the larger grain size.

Matching light capture and energy demand is especially relevant for plants subjected to stressful growth conditions (Lawlor and Tezara, 2009). The fact that photosynthesis is a major energy demanding process implies that in cases where photosynthesis is impaired and light excitation energy is in excess, leaves might suffer photooxidative damage. The excessive excitation energy in photosystem II (PSII) will lead to an impairment of photosynthetic function, progress to an accumulation of reactive oxygen species (ROS), and thereby result in oxidative stress. Changes in chlorophyll fluorescence parameters for dark-adapted leaves are expected under severe drought or heat stress, such as increases in Fo and decreases in Fm and Fv/Fm (Roostaei et al., 2011). In contrast, the value of Fv/Fm for non-stressed leaves is remarkably consistent at ∼0.75 (Björkman and Demmig, 1987). In our experiment Fm and Fv/Fm were not different between the two cereals and among environments, except at anthesis for Fv/Fm (Table 3), and this was probably because the plants were not exposed to severe water stress. The higher ETRmax of triticale compared to wheat (12.2 and 21.4% at anthesis and grain filling, respectively) and Y(II) (11.8 and 23.1% at anthesis and grain filling, respectively) is consistent with the higher An observed at anthesis (Tables 2, 4). Y(II) corresponds to the fraction of energy that is photochemically converted in PSII (Klughammer and Schreiber, 2008; Scherner et al., 2013), therefore triticale shows a better PSII capacity (Sharma et al., 2015). However, triticale had a lower NPQ compared to wheat (19.5%), indicating that wheat may be able to better regulate non-photochemical energy dissipation (Sofo, 2011), at the expense of photosynthesis.

### Relationships Between Physiological and Agronomic Traits

Δ^13^C was positively correlated with GY and HI, but the correlation was higher with HI (Table 7), and this could reflect the efficiency of carbon partitioning to the kernel (Merah et al., 2001). During grain growth, most of the assimilated come from photosynthesis in post-anthesis (Munjonji et al., 2017); in the case of triticale, the higher An, associated to greater light capture (higher biomass) and grains/ear may be driving the higher yields (Roques et al., 2017).

According to Motzo et al. (2013), the yield potential of triticale was associated to greater stomatal conductance, nevertheless in the present research wheat presented higher gs, but also used more energy in photoprotection [Y(NPQ) and NPQ] than triticale (Figure 4 and Table 4); this could explain, at least in part, the lower GY of wheat, particularly under water-limited conditions. The PCA indicates that in more stressful situations, the differences between species were mainly determined by physiological traits (and TKW), especially the functioning of the photosynthetic apparatus, whereas in less stressful conditions, with fewer limitations for the assimilation of CO_2_, the differences between species are determined for its productivity.

Thousand kernel weight presents in general high heritability and is less affected by the water deficit (Arguello et al., 2016; Mathew et al., 2018). In triticale, the significant correlation between TKW and gas exchange (An, gs, An/Ci), and the higher chlorophyll content compared to wheat, suggest that triticale continue assimilating carbon and mobilizing it to the grain, during grain filling.

## Conclusion

In field conditions under different environments and water regimes, triticale (cv. Aguacero) has usually higher GY associated with larger numbers of grains per spike and larger kernel weight compared to bread wheat. In terms of tolerance to water deficit, triticale appeared to be more susceptible than wheat, because the reduction of GY under severe water stress compared to well-watered condition. The physiological characterization indicated that the two species have different strategies; triticale presented higher photosynthetic rates that contributed to increase plant growth and yield in the different environments, whereas wheat showed higher photoprotection system in detriment of assimilate production. Regardless of the water treatment, the contribution of post-anthesis CO_2_ assimilation to grain filling was higher in triticale. In fact, TKW was positively correlated with An and ETRmax in triticale. The higher An in triticale was linked to improved water use and apparent carboxilation efficiency.

## Author Contributions

AM-E, SR-B, FE, and MG performed the evaluations of physiological traits. DC and IM was in charge of the management of field experiments and evaluation of agronomic traits. AdP, AM-E, GL, and IA performed the data analysis. AdP was in charge of the writing up but all the authors contributed to the manuscript.

## Conflict of Interest Statement

The authors declare that the research was conducted in the absence of any commercial or financial relationships that could be construed as a potential conflict of interest.

## Supplementary Material

FIGURE S1 Principal component analysis (PCA) of physiological and agronomic traits determined in triticale (Trit) and wheat (Wh) in different environments. Symbols represent the combination of species, water regime (WW, well-watered; WL, water-limited), site (CAU, Cauquenes; SR, Santa Rosa), and year (2015 and 2106). In **(A)** biplot of the first two principal components (PC1 and PC2) and the position of the two species on the different environments; in **(B)** the loading-plots of physiological and productivity traits measurements used in the PCA.
