# Supplementary figures and images for: Exploring Agronomic and Physiological Traits Associated With the Differences in Productivity Between Triticale and Bread Wheat in Mediterranean Environments
Source: Front Plant Sci. 2019 Apr 5;10:404. doi: 10.3389/fpls.2019.00404 (PMC6460938; doi:10.3389/fpls.2019.00404)

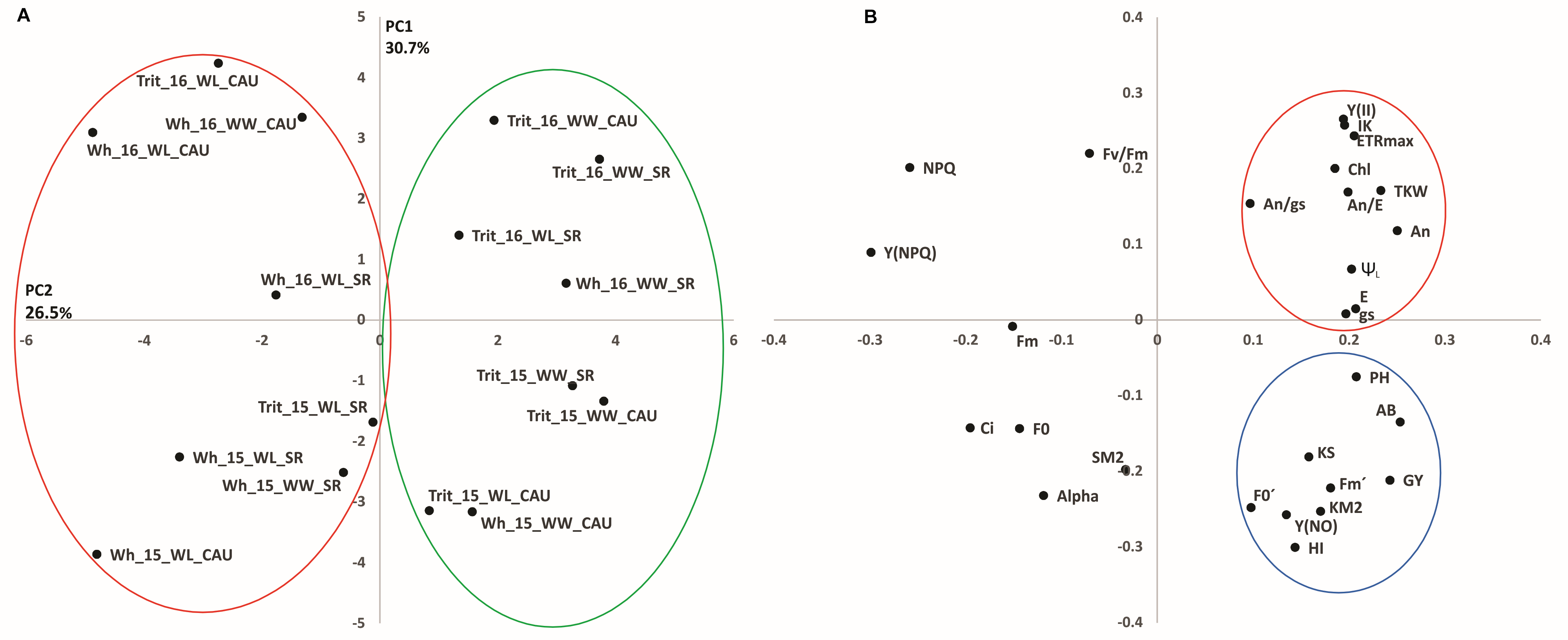

Supplement: FIGURE S1 — Principal component analysis (PCA) of physiological and agronomic traits determined in triticale (Trit) and wheat (Wh) in different environments. Symbols represent the combination of species, water regime (WW, well-watered; WL, water-limited), site (CAU, Cauquenes; SR, Santa Rosa), and year (2015 and 2106). In (A) biplot of the first two principal components (PC1 and PC2) and the position of the two species on the different environments; in (B) the loading-plots of physiological and productivity traits measurements used in the PCA. [file Image_1.tif]
